# Characteristic and fate determination of adipose precursors during adipose tissue remodeling

**DOI:** 10.1186/s13619-023-00157-8

**Published:** 2023-05-04

**Authors:** Jiayin Ye, Cheng Gao, Yong Liang, Zongliu Hou, Yufang Shi, Ying Wang

**Affiliations:** 1 CAS Key Laboratory of Tissue Microenvironment and Tumor, Shanghai Institute of Nutrition and Health, University of Chinese Academy of Sciences, Chinese Academy of Sciences, 320 Yueyang Road, Shanghai, 200031 China; 2 Key Laboratory of Tumor Immunological Prevention and Treatment of Yunnan Province, Kunming, 650000 Yunnan China; 3 The Third Affiliated Hospital of Soochow University and State Key Laboratory of Radiation Medicine and Protection, Institutes for Translational Medicine, Soochow University, 199 Renai Road, Suzhou, 215123 Jiangsu China

**Keywords:** Adipose precursors, Inflammation, Adipose tissue remodeling, Metabolic disorders

## Abstract

Adipose tissues are essential for actively regulating systemic energy balance, glucose homeostasis, immune responses, reproduction, and longevity. Adipocytes maintain dynamic metabolic needs and possess heterogeneity in energy storage and supply. Overexpansion of adipose tissue, especially the visceral type, is a high risk for diabetes and other metabolic diseases. Changes in adipocytes, hypertrophy or hyperplasia, contribute to the remodeling of obese adipose tissues, accompanied by abundant immune cell accumulation, decreased angiogenesis, and aberrant extracellular matrix deposition. The process and mechanism of adipogenesis are well known, however, adipose precursors and their fate decision are only being defined with recent information available to decipher how adipose tissues generate, maintain, and remodel. Here, we discuss the key findings that identify adipose precursors phenotypically, with special emphasis on the intrinsic and extrinsic signals in instructing and regulating the fate of adipose precursors under pathophysiological conditions. We hope that the information in this review lead to novel therapeutic strategies to combat obesity and related metabolic diseases.

## Background

The discovery of lipid storage from prokaryotes to humans highlights that such a highly conserved evolutionary process is to originally adapt for energy demands under unfavorable conditions. Lipids are also important constituents for membrane biosynthesis and for cellular signaling. Starting with arthropods, there are specialized adipocytes for lipid storage. In vertebrates, there is a committed tissue for fat storage, called adipose tissue (Birsoy et al. 2013). In fact, adipocytes belong to two functional and histological lineages: white adipose tissue (WAT) to store energy, and brown adipose tissue (BAT) to dissipate energy (Cinti 2018).

In mammals, it is generally accepted that adipocytes fall into three main categories, including white adipocytes, brown adipocytes, and beige adipocytes (Table 1) (Cohen and Kajimura 2021; Sakers et al. 2022; Sebo and Rodeheffer 2019). Among them, white adipocytes are featured by a large unilocular lipid droplet (LD) and few mitochondria, which are mainly responsible for energy storage. Brown and beige adipocytes, on the other hand, are enriched with multilocular LDs and a large number of mitochondria, which are involved in energy consumption and heat production. A big difference between beige adipocytes and brown adipocytes is that they reside in adipose tissue depots (Wu et al. 2012), i.e., brown adipocytes locate in BAT, while beige adipocytes reside in WAT. They are generated from distinct precursors, which will be eluted below. Nevertheless, the difference of these adipocyte locations confers the adipose tissue specificity to meet the diverse needs of the body under different conditions.

**Table 1:**
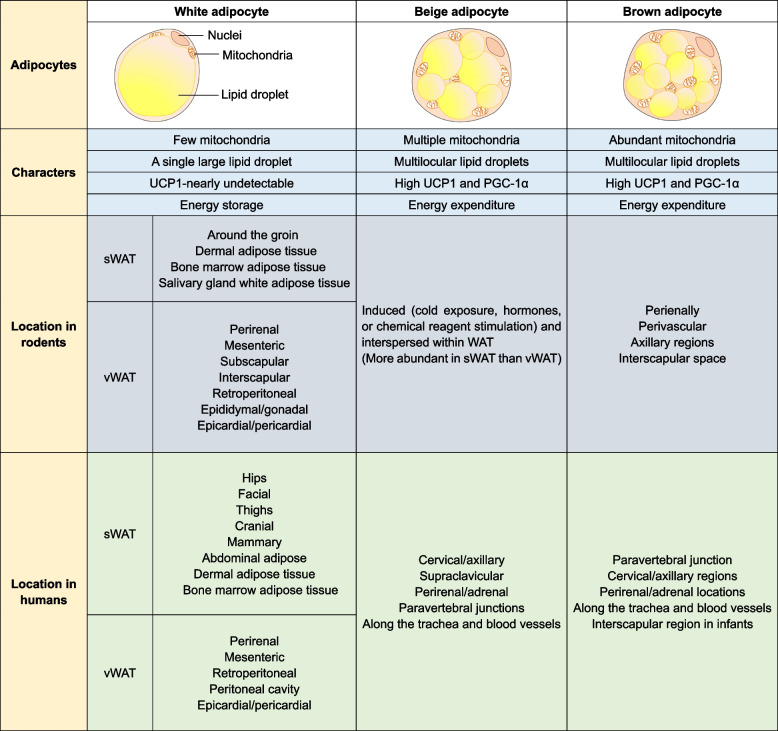
The features of adipose tissue in rodents and humans

The table outlines the characteristics and distribution of different adipose tissues in rodents and humans

*sWAT* Subcutaneous white adipose tissue, *vWAT* Visceral white adipose tissue, *UCP1* Uncoupling protein1, *PGC-1α* Peroxisome proliferator-activated receptor γ coactivator 1α

These adipocytes, together with immune cells, mesenchymal stem cells (MSCs), pericytes, endothelial cells, nerves, and fibroblasts, form the adipose tissue (Peirce et al. 2014). In addition to regulating energy homeostasis, adipose tissue is also an endocrine and immune tissue, with the abilities to maintain body temperature and to regulate metabolism homeostasis, immune responses, reproduction, as well as longevity (Rosen and Spiegelman 2014). Immoderate and insufficient fat storage are related to various disease conditions, such as insulin resistance, diabetes, hyperlipidemia, cardiovascular disease, emphasizing the pivotal role of adipose tissue (Stenkula and Erlanson-Albertsson 2018). During obesity, the expansion of WAT to facilitate fat storage is related to adipocyte hypertrophy, a process characterized by increased adipocyte size, and adipocyte hyperplasia, highlighting the generation of new adipocytes in cell number (Gupta 2014). Meanwhile, the obese adipose tissue possesses abundant immune cells, reduced angiogenesis, and excessive extracellular matrix (ECM) deposition (Hammarstedt et al. 2018; Quail and Dannenberg 2019).

For decades, biologists have dedicated to identify adipose stem cells or adipose precursors and to understand how adipogenesis can be regulated to meet the energy demand of the body (Marcelin et al. 2017). However, adipose precursors are quite diverse and present a spatial and temporal heterogeneity in response to a number of pathophysiological or pharmacological conditions, such as changes in temperature and metabolites, signals from immune cells, and stimulation of cytokines and hormones (Ghaben and Scherer 2019; Pyrina et al. 2020). Single cell RNA-sequencing (scRNA-seq) analysis on the inguinal WAT of mice with the treatment of β3-adrenaline receptor agonist, a method to mimic cold exposure and induce the generation of brown adipocytes, revealed that distinct populations of adipose stem cells possess different potential to enter the adipogenic pathway (Burl et al. 2018). More importantly, the generation of new adipocytes, regardless of white, beige, or brown, from adipose precursors is pivotal in helping adipose tissue to adapt to changes in energy balance and protect against metabolic diseases. Here, we focus on the identification and regulation of adipose precursors on metabolic events and discuss potential strategies to manipulate fat in order to fight obesity and related metabolic disorders.

## Origin of adipocytes and their precursors identification

Adipocytes are generated from MSCs, a population of multipotent progenitors believed to be derived from mesoderm (Chen et al. 2016). Recent investigations also revealed that some adipocytes originate from the ectodermal neural crest (Fu et al. 2019). During the infancy and adolescence, expansion of adipose tissue is attributed to the increase in the size and number of adipocytes. The latter is controlled by adipose stem cells. It is well-known that adipose stem cells undergo two phases, the commitment phase to adipose precursors and the differentiation phase to generate adipocytes. Adipose precursors include adipose progenitors and preadipocytes. According to the different marker expression, they show the hierarchy in vivo during adipogenesis (Berry et al. 2014). However, the questions on the self-renewal and repopulation of adipose progenitors remain unclear. Therefore, if not specified, adipose precursors represent adipose progenitors and preadipocytes. With respect to adipose tissue development and its remodeling during obesity, new adipocyte generation from adipose stem cells or progenitors is the key in controlling the homeostasis of adipose tissue (Kim et al. 2007).

### Origin of adipocytes at the prenatal stage

During gastrulation, mesoderm gradually forms along with the migration of endoderm and ectoderm cells. It is well established that the adipose tissue closely associated with the tissues and organs developed from the mesoderm has a common origin (Zhai et al. 2022). By establishing a constitutively active *Wt1-Cre;R26RYFP* mouse model to trace Wilm tumor gene 1 positive (Wt1+) cells and their progeny, it has been demonstrated that most of adipocytes in the visceral WAT (vWAT) are originated from the lateral mesoderm mesothelial cells expressing Wt1 during the embryonic stage, while these Wt1 cells were not enriched in subcutaneous WAT (sWAT) and BAT (Chau et al. 2014). This study is the first to directly demonstrate that visceral adipocytes originate from the mesoderm, and also reveals an ontogenetic difference among distinct fat depots (Chau et al. 2014). Subsequent studies also confirmed that adipocytes originate from the mesoderm, however, adipose progenitor phenotypes vary, for example, myogenic factor 5 positive or negative (Myf5+ or Myf5-) progenitor cells in the paraxial mesoderm (Sanchez-Gurmaches et al. 2012), Wt1+ mesothelial cells (Chau et al. 2014), and preadipocyte factor 1 positive (Pref-1+) cells with mesenchymal properties at the early embryonic stage (Gulyaeva et al. 2019). When the fate of ectodermal neural crest cells was mapped in vivo using SRY-box transcription factor 10 (Sox10) tracing mice, these neural crest cells were verified to be able to generate adipocytes in the sWAT located in the area from the salivary gland to the ear region (Billon et al. 2007; Schoettl et al. 2018). Lineage tracing of Wnt family member 1 (Wnt1), which is specifically expressed in the neural crest, also found that craniofacial adipocytes are derived from ectoderm. However, Wnt1 is not detected either in vWAT or sWAT (Fu et al. 2019). In addition, neuroectoderm is a main source of adipose precursors in head adipose tissue, as a part of cephalogenesis, but along with aging, the proportion of these neural crest-derived adipose precursors decreases and are replaced by cells of yet to be defined origin, indicating that the origin of adipocytes during development could shift dynamically (Sowa et al. 2013). Unlike sWAT in the head, its depot in the trunk and limb, except male perigonadal depot originates from the posterior portion of lateral plate mesoderm (Sebo et al. 2018).

### Identification of adipose precursors in the adipose tissue homeostasis

In adults, the number of adipocytes is relatively stable, with around 10% of adipocytes renewed every year, and thus adipose precursors are pivotal in maintaining adipose tissue homeostasis (Spalding et al. 2008). CD24 has been used to identify adipose precursors in *vivo*. Its deficiency in mice led to a reduction in the mass of sWAT and vWAT around 40% to 74%. Meanwhile, these mice exhibited the enhancement in fasting glucose and free fatty acids, and the reduction of fasting insulin and leptin in blood (Fairbridge et al. 2015). According to the key role of peroxisome proliferator-activated receptor (PPAR)-α and PPARγ in regulating the adipogenesis, mice with *Ppara* deficiency have a marked suppression on BAT in response to cold exposure, concurrent with a prominent decrease in fatty acid oxidation and energy consumption (Hondares et al. 2011; Tong et al. 2005). In addition, mice with PPARγ agonist treatment increase the expression of thermogenic genes, insulin sensitivity, and energy expenditure (Rachid et al. 2015). These results suggest the crucial role of in vivo adipogenesis in maintaining energy balance.

Adipose precursors are enriched in the stromal vascular fractions (SVF), which is identified by a panel of markers, being positive in CD29, CD34, stem cell antigen-1 (Sca-1), and CD24, as well as negative in CD45, CD31, and Ter119 (Rodeheffer et al. 2008). The formation of adipocytes occurs in the close vicinity of vascular endothelium, further supporting that the adipose precursors are located adjacent to blood vessel (Gupta et al. 2012). This also raises a question that pericytes, a kind of mural cells that distribute along the capillary marked by smooth muscle α-actin (αSMA), NG2 proteoglycan, as well as platelet-derived growth factor receptor β (PDGFRβ), may be a vital source for adipocytes. In *Pdgfrb-Cre* mediated lineage tracing studies, adipocytes in retroperitoneal and inguinal adipose tissues, but not in other locations, originate from PDGFRβ+ pericytes (Tang et al. 2008). Of note, the commitment of white adipocyte precursors has been assigned prenatally or at the early stage of postnatal development (Tang et al. 2008). Through establishing knock-in mice expressing full length PDGFRβ or PDGFRβ with mutations to allow its constitutive activation, a comparable postnatal adipose tissue generation was seen, showing that pericytes are not the only origin of adipocytes. Although elevated PDGFRβ signaling in adipose precursors, regardless of its expression in pericytes or other mesenchymal cells, activation of PDGFRβ inhibits white adipocyte differentiation (Olson and Soriano 2011).

Unlike abundant brown fat deposits in rodents and other smaller mammals, BAT in larger mammals almost diminishes after infancy. Interestingly, BAT and skeletal muscle share the same origin (Atit et al. 2006; Billon and Dani 2012). It was found that both of them are originated from Myf5+ progenitor cells (Seale et al. 2008). Profiling the transcription and mitochondrial proteomics also demonstrated the kinship between brown adipocytes and myocytes (Forner et al. 2009; Timmons et al. 2007). In particular, the development and differentiation of skeletal muscle and BAT are competing reciprocally. When PR domain-containing protein 16 (PRDM16), a gene related to muscle formation, was knocked out in mice, BAT generation was decreased, and muscle formation was increased (Seale et al. 2008). Thus, there is a distinct origin for brown adipocytes and white adipocytes.

**Fig. 1:**
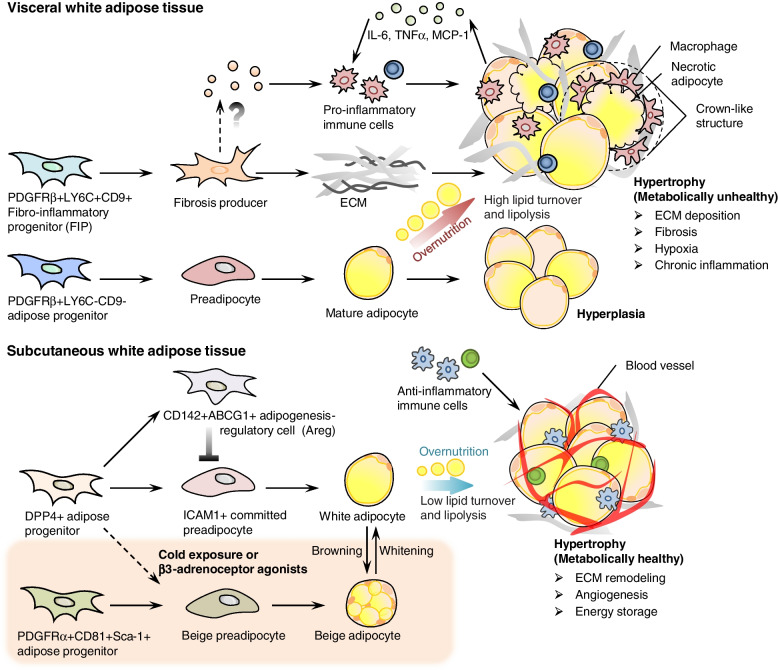
Heterogeneity and plasticity of adipose precursors in white and beige adipogenesis. Adipogenesis is a process involving two phases, lineage commitment and terminal differentiation. The former is the commitment of adipose stem cells to adipose precursors. The latter is terminal differentiation of adipose precursors to matured adipocytes. Interestingly, the precursors in different WAT depots have complex populations and characteristics. In vWAT, there are two subpopulations of PDGFRβ+ progenitors, LY6C-CD9- and LY6C+ populations. Among them, LY6C-CD9-PDGFRβ+ cells are adipose progenitors, with the ability of commitment to white adipocytes. LY6C+ PDGFRβ+ progenitors representing FIPs that have lost the ability to differentiate into adipocytes, but gain the ability to promote tissue inflammation and fibrosis. Using scRNA-seq, DPP4+ progenitor cells in sWAT can be converted to ICAM1+ committed preadipocytes, and CD142+ ABCG1+ Aregs that act as brake in adipogenesis. During obesity in rodents, the remodeling of vWAT is attributed to hyperplasia and hypertrophy, while the expansion of sWAT is mainly relied on hypertrophy. Importantly, the excessive expansion in vWAT is closely linked to metabolically unhealthy obesity, whereas sWAT expansion to certain extend is expected to improve lipid storage stress and metabolic status. In addition, the PDGFRα+ CD81+ beige adipose precursors existing in sWAT contribute to the de novo beige adipogenesis upon long-term cold exposure and β3-adrenergic receptor activation. Meanwhile, short-term stimuli can convert mature white adipocytes into beige adipocytes. These browning strategies hold the promise to combat obesity and related metabolic disorders

Pref-1 is also a marker for adipose precursors. It appears in the mesenchymal portion of the mouse back at E10.5, but not endothelial or pericytal (Hudak et al. 2014). At E17.5, adipocytes containing LDs can be observed on the back and gradually develop into sWAT (Hudak et al. 2014). It is well-established that Pref-1+ cells can differentiate into white adipocytes, and Pref-1 expression gradually decreases during the adipogenesis process, suggesting that Pref-1 marks adipose precursors (Gulyaeva et al. 2018). A lineage hierarchy composed of several distinct types of mesenchymal cells in the adipose tissue was revealed by scRNA-seq and cell trajectory analysis (Merrick et al. 2019). Dipeptidyl peptidase-4 positive (DPP4+) progenitor cells in the reticular interstitium of sWAT can generate intercellular cell adhesion molecule-1 positive (ICAM-1+) and CD142+ preadipocytes (Fig. 1). Among them, ICAM-1+ preadipocytes featured with *Pref-1* and *Pparg* expression exist in the adipose tissue of humans and mice, and differentiate into mature white adipocytes. In human adipose tissues, ICAM-1+ and CD142+ /*Clec11a*+ preadipocytes are in the same population, however, there are in different subpopulations of preadipocytes in mice (Merrick et al. 2019). Interestingly, Schwalie et al. found that CD142+ / ATP binding cassette subfamily G member 1-expressing (ABCG1+) stromal cells were a group of adipogenesis-regulatory cells (Aregs), which showed the anti-adipogenic activity (Schwalie et al. 2018). Nevertheless, there is a hierarchy in the process of adipose precursors in generating white adipocytes in vivo.

The heterogeneity of adipose precursors can be embodied by their ability in generating distinct types of adipocytes and by the sites they reside. In vivo fate mapping of adipocytes demonstrated that Myf5+ adipose precursors, sharing the properties with myogenic lineage, can give rise to brown adipocytes, but not beige and white adipocytes (Seale et al. 2008). Detailed analysis further showed that Myf5 precursors are not the exclusive source of brown adipocytes and white adipocytes in interscapular and retroperitoneal WAT arise from Myf5+ cells, while adipocytes in inguinal and gonadal WAT are mostly derived from Myf5- cells (Sanchez-Gurmaches and Guertin 2014; Sanchez-Gurmaches et al. 2012). However, it remains elusive if white adipocytes generated from Myf5+ progenitor cells present different functions with cells in vWAT and sWAT, showing the abilities alike to beige or brown adipocytes. A population of adipose precursors to generate beige adipocytes was found in the sWAT and featured by the expression of CD137 and transmembrane protein 26 (TMEM26) (Wu et al. 2012). Upon cold exposure or β3-agonist stimulation, the newly generated beige adipocytes are generated from CD137-expressing progenitor cells (Wang et al. 2013; Wu et al. 2012). In addition, large-scale genetic analysis suggested that beige adipocytes in sWAT of the groin and axilla had high expression of genes related to smooth muscle-like cells, such as *Acta2*, *Tagln*, *Myh11*, *Myl9*, and *Cnn1* (Long et al. 2014). Tracing these smooth muscle-like cells also showed that they can generate beige adipocytes upon cold exposure (Long et al. 2014). However, smooth muscle cells have various origins, and whether beige adipocytes are differentiated from one of the populations remains to be further studied (Kajimura et al. 2015). Overall, advanced progression in identifying adipose precursors and their lineage trajectory in generating functionally distinct adipocytes can help us to understand the development and remodel of adipose tissue under distinct pathophysiological conditions (Table 2).

**Table 2:** The heterogeneity of adipose precursors revealed by scRNA-seq analysis

Author	Species	Sample	Pre-processing	Population	Markers	Definition or function
Merrick et al (Merrick et al*.* 2019)	Mouse	IngWAT	CD45-	Group 1	*Dpp4*+ *, Wnt2*+ *, Bmp7*+ *, Pi16*+	Interstitial progenitors (produce ICAM1+ and CD142+ preadipocytes)
				Group 2	*Icam1*+ *, Pref1*+ *, Pparg*+ *, Fabp4*+ *, Cd36*+	Committed preadipocytes
				Group 3	*Cd142*+ *, Clec11a*+ *, Fmo2*+	Another preadipocytes
	Human obese	sWAT	CD45-	Group 1	*DPP4*+ *, CD55*+ *, WNT2*+	Comparable to murine group 1 cells
				Group 2	*ICAM1*+ *, PPARG*+ *, GGT5*+	Comparable to murine group 2 cells and group 3 cells
Vijay et al (Vijay et al. 2020)	Human obese	sWAT	CD45- CD34+ CD31-	SP1 and SP3	*MGP*+ *, APOD*+ *, CXCL14*+ *, WISP2*+	Preadipocytes/adipose stem cells
				SP2	*APOE*+ *, FABP4*+ *, CEBPB*+ *, CD36*+	More mature adipocytes progenitor cells
				SP4	*COL3A1*+ *, COL6A3*+ *, COL1A1*+ *, COL6A1*+	Fibrosis and extra cellular matrix accumulation
		vWAT	CD45- CD34+ CD31-	VP4	*APOD*+ *, CXCL14*+ *, DPT*+ *, GPX3*+ *, MGP*+ *, EIF1*+ *, C1S*+ *and ADH1B*+	Similar to SP1 and SP3
				VP5	*MFAP5*+ *, S100A4*+	Adipose tissue remodeling; inhibition of adipogenesis
Oguri et al (Oguri et al*.* 2020)	Mouse	iBAT, IngWAT, EpiWAT	Lin- stromal cells	Types I	*Pdgfra*+ *, Akt2*+ *, Wnt4*+	Not mentioned
				Type II	*Pdgfrb*+ *, Cspg4*+ *, Ednrb*+	An endothelial signature
				Type III	*Cd81*+ *, Pdgfra*+ *, Sm22*+ *, Acta2*+	From IngWAT; contribute to de novo beige adipogenesis
				Types IV	*Pdgfra*+ *, Mmp3*+	Not mentioned
				Types V	*Pdgfra*+ *, Atf6*+ *, Acot2*+	Not mentioned
Emont et al (Emont et al. 2022)	Mouse	IngWAT and EpiWAT of mice fed ND or HFD	stromal vascular fractions	mASPC1	*Pdgfra*+ *, Pde11a*+	Not mentioned
				mASPC2	*Pdgfra*+ *, Aldh1a3*+	Early progenitor cells; more abundant in IngWAT than EpiWAT; differentiate into mASPC6 upon HFD
				mASPC3	*Pdgfra*+ *, Mgp*+	Not mentioned
				mASPC4	*Pdgfra*+ *, Epha3*+	Not mentioned
				mASPC5	*Pdgfra*+ *, Tenm3*+	More prevalent in EpiWAT versus IngWAT
				mASPC6	*Pdgfra*+ *, Frem1*+	More prevalent in EpiWAT versus IngWAT, increase markedly in response to HFD in PG
	Human	sWAT and vWAT		hASPC1	*PDGFRA*+ *, CEBPD*+	More prevalent in sWAT than vWAT
				hASPC2	*PDGFRA*+ *, ALDH1A3*+	Not mentioned
				hASPC3	*PDGFRA*+ *, FGF10*+	More abundant in vWAT
				hASPC4	*PDGFRA*+ *, EPHA3*+	More abundant in sWAT than vWAT; increases in sWAT in subjects with higher BMI
				hASPC5	*PDGFRA*+ *, SGCZ*+	More abundant in sWAT than vWAT; increases in sWAT in subjects with higher BMI
				hASPC6	*PDGFRA*+ *, PDE4D*+	More prevalent in vWAT
Burl et al (Burl et al. 2018)	Mouse	IngWAT and EpiWAT of control and CL316243 (β3-adrenoceptor agonists)-treated mice	Lin- stromal cells	ASC1	*Pdgfra*+ *, Sca1*+ *, Icam1*+ *, Col4a2*+	Adipocyte stem cell group 1
				ASC2	*Pdgfra*+ *, Sca1*+ *, Pi16*+ *, Dpp4*+	Adipocyte stem cell group 2; more prevalent in IngWAT
				Diff.ASC	*Plin1*+ *, Car3*+ *, Cebpa*+	Differentiating adipocyte stem cells; CL316243-induced clusters in EpiWAT
				Pro.ASC	Genes that positively regulate cell cycle	Proliferating adipocyte stem cells; CL316243-induced new clusters in IngWAT and EpiWAT
Hepler et al (Hepler et al. 2018)	Mouse	EpiWAT	PDGFRβ+	APCs	*Ly6c-, Cd9-*	Adipocyte precursor cells
				FIPs	*Ly6c*+ *, Cd9*+	Fibro-inflammatory progenitors
				MLCs	*Ly6c*+ *, Cd9-*	Mesothelial-like cells
Schwalie et al (Schwalie et al. 2018)	Mouse	IngWAT	Lin- CD29+ CD34+ Sca1+	G1/G4 (P1)	*Cd55*+ *, Il13ra*+	Adipose stem cells
				G2 (P2)	*Vap1*+ *, Adam12*+	Preadipocytes
				G3 (P3)	*Cd142*+ *, Abcg1*+	Adipogenesis-regulatory cells; inhibit adipogenesis
Cho et al (Cho et al. 2019)	Mouse	EpiWAT of mice fed ND or HFD	Sca1+ CD45- CD31- Ter119-	Cluster1	CD55^high^/CD81^low^	More primitive, undifferentiated ASCs
				Cluster5	CD55^high^/CD81^high^	More primitive, undifferentiated ASCs
				Cluster2	CD55^low^/CD9^low−mid^/CD81^low^	Committed preadipocytes
				Cluster4	CD55^low^/CD9^low−mid^/CD81^high^	Committed preadipocytes
				Cluster6	CD55^low^/CD9^high^	Committed preadipocytes
Nahmgoong et al (Nahmgoong et al. 2022)	Mouse	IngWAT and EpiWAT of mice fed ND or HFD	Lin- (CD31- and CD45-) stromal vascular fractions	E_N_1 and I_N_1–I_N_4	*Dpp4*+	Multipotent ASCs expressing BMPs
				E_N_2–E_N_3 and I_N_5	*Dpp4*+ *, Icam1*+ *, Cebpd*+	Early committed preadipocytes
				E_N_4 and I_N_6–I_N_7	*Icam1*+ *, Fabp4*+	Late committed preadipocytes

The table summarizes the populations of adipose precursors obtained by scRNA-seq analysis

*sWAT* Subcutaneous white adipose tissue, *vWAT* Visceral white adipose tissue, *iBAT* Interscapular brown adipose tissue, *IngWAT* Inguinal white adipose tissue, *EpiWAT* Epididymal white adipose tissue, *PG* Perigonadal adipose tissue, *SP* sWAT progenitor, *VP* vWAT progenitor, *ASPC* Adipose stem and progenitor cell, *ASCs* Adipocyte stem cells, *APCs* Adipocyte precursor cells, *FIPs* Fibro-inflammatory progenitors, *MLCs* Mesothelial-like cells, *ND* Normal diet, *HFD* High fat diet

## Intrinsic signals controlling adipogenesis

The adipogenesis of adipose stem cells has been artificially divided into two phases, the determination phase which relates the commitment of multipotent stem cells to the adipocyte lineage, and the terminal differentiation phase, a process during which adipose precursors differentiate into adipocytes (Ghaben and Scherer 2019). The two phases are involved in the white, brown and beige adipogenesis.

### White adipocyte differentiation

Due to the heterogeneous properties of adipose precursors in vivo (Table 2), most understandings on the signaling pathways controlling adipogenesis are derived in vitro experiments. Cell lines, 3T3-L1 from Swiss 3T3 mouse embryos, C3H10T1/2 from C3H mouse embryos, LiSa-2 from a poorly differentiated pleomorphic liposarcoma (Wabitsch et al. 2000) and SGBS from subcutaneous SVF of an infant with Simpson-Golabi-Behmel syndrome (Wabitsch et al. 2001), are often used to study the mechanisms of adipogenesis. Using C3H10T1/2 as a multipotent stem cell model, bone morphogenesis proteins, bone morphogenetic protein (BMP) 2 and 4, were found to induce the commitment of these cells to differentiate into adipocytes through the BMP receptor and the SMAD family member 4 (SMAD4) signaling process (Huang et al. 2009). Notably, transcriptional factors of early b-cell factor 1 (EBF1) and zinc finger protein 423 (ZFP423) are enriched in committed precursors and at the same time increased the sensitivity of these cells to BMP signaling (Gupta et al. 2010; Jimenez et al. 2007). In contrast, ZFP521, a transcriptional factor related to the Wnt signaling pathway, forms a complex with EBF1, inhibits the transcription of ZFP423, and thereby impairs adipogenesis (Kang et al. 2012). After lineage commitment to adipose precursors, these cells eventually form adipocytes under defined adipogenesis conditions (Ghaben and Scherer 2019). During in vitro white adipocyte differentiation, the cascade activity of cAMP-response element-binding protein (CREB), CCAAT/enhancer-binding protein (C/EBP)-β, and C/EBPδ transcription forms a dominant pathway (Zhang et al. 2004). Activation of C/EBPβ triggers the activation of PPARγ and C/EBPα, which have been shown to initiate adipogenesis (Tang and Lane 2012; Tang et al. 2003). PPARγ synergized with C/EBPα to activate several genes related to adipocyte generation (Hammarstedt et al. 2018). However, it has been reported that C/EBPα is dispensable for in vivo embryonic adipogenesis (Wang et al. 2015). Distinct from mouse adipose precursors that undergo mitotic clonal expansion at the early phase of white adipogenesis in vitro (Audano et al. 2022), primary adipose precursors derived from human adipose tissue directly enter the terminal differentiation phase in vitro, without mitotic clonal expansion (Entenmann and Hauner 1996). In addition, except for LiSa-2, the culture medium for the adipogenesis of human adipose precursors needs the addition of T3 supplementation and PPAR agonists, such as rosiglitazone, indicating that these cells may lack endogenous PPARγ ligands compared to rodent cells (Hauner et al. 1989; Dufau et al. 2021; Tzameli et al. 2004). Indeed, studies have shown that in vivo PPARγ ligands are produced by microvascular endothelial cells of human WAT (Gogg et al. 2019; Gustafson and Smith 2012). Nevertheless, the overall regulatory mechanisms of adipogenesis between mouse and human are highly conserved (Schmidt et al. 2011). Thus, diverse spatiotemporal transcription activities during adipogenesis must occur in vivo.

### Brown and beige adipocyte differentiation

During the early stages of brown adipogenesis, EBF2 acts as a major factor in dictating the commitment of brown adipose precursors. Meanwhile, the interaction between Ewing sarcoma breakpoint region 1 (EWSR1; also known as EWS) and Y-box-binding protein 1 (YBX1; also known as NSEP1) stimulates the transcription of BMP7 and facilitates the commitment of brown adipocytes. During the terminal differentiation process, EBF2 combined with PPARγ promotes the brown adipocyte differentiation and enhances the activity of PRDM16 and PPARγ coactivator 1α (PGC-1α), driving the thermogenesis program (Wang and Seale 2016). Also, PRDM16 can interact with C/EBPβ, PPARγ, and ZFP516 to promote the terminal differentiation of brown adipocytes. In addition, interferon regulatory factor 4 (IRF4) was found to regulate the expression of thermogenic genes via acting as a partner of PGC-1α (Gupta 2014). Although the precursors are different in giving rise to brown adipocytes and beige adipocytes, their adipogenesis process and signaling pathways involved in thermogenesis are similar. In response to β3-norepinephrine agonist, white adipose precursors enter the beige adipogenesis progress through promoting the expression of thermogenic genes in dependence of PPARγ and EBF2. This process can be suppressed by transcriptional factors related to white adipogenesis. Indeed, using CRISPR-Cas9 editing to disrupt the interaction between ZFP423-EBF2 induced extensive “browning.” When ZFP423 was lost in adipocytes, an EBF2 NuRD-to-BAF coregulator switched and a shift of PPARγ occupancy to thermogenic genes formed (Shao et al. 2016; Shao et al. 2021b), providing a potential therapeutic target for fighting obesity.

Two ways are always emphasized while analyzing the generation of beige adipocytes. They are de novo differentiation of beige adipocytes from adipose precursors and browning of white adipocytes (Fig. 1). Using Adipo-Chaser mouse, newly generated beige adipocytes stem from de novo differentiated adipocytes during the browning of sWAT induced by cold exposure, excluding the possibility of transforming from white adipocytes to beige adipocytes (Wang et al. 2013). However, the de novo beige adipogenesis needed a long-term cold exposure, when these PDGFRβ+ mural cells were monitored (Vishvanath et al. 2016). A more elegant study using Dre and Cre recombinase-mediated intersectional genetic mouse model showed that PDGFRα+ PDGFRβ+ perivascular mesenchymal cells are the source for generating beige adipocytes under cold exposure. Also, pre-existing adipocytes can be converted to beige adipocytes (Han et al. 2021). When mice are deficient in stearoyl-CoA desaturase 1 (SCD1), a rate-limiting enzyme in catalyzing saturated fatty acids into monounsaturated ones, there is an increase in white adipose precursors derived beige adipocytes, but not the browning of white adipocytes (Liu et al. 2020). Such a dramatic change is attributed to the accumulation of succinate in adipose precursors, which fuels the mitochondrial complex II and determines the beige biogenesis.

## Extrinsic signals regulating adipogenesis

Apart from the complex intracellular signaling in controlling the fate of adipose precursors, the advent of in situ technology and single cell analysis for the study of tissue microenvironment has shown that extrinsic factors can signal specific information to adipose precursors and determine their differentiation potential during development and remodeling of adipose tissue under various pathophysiological conditions. These extrinsic factors include hormones, inflammatory factors, ECM, and circadian rhythms. As the regulation of hormone and circadian rhythm in adipogenesis is well discussed in previous literatures (Bahrami-Nejad et al. 2018; Cohen and Kajimura 2021; Ghaben and Scherer 2019; Lee 2017; Luchetti et al. 2014), in this review, we focus on the influence of inflammation and ECM on adipose tissue remodeling.

### The role of inflammation in regulating adipogenesis

In 1993, the discovery of enhanced tumor necrosis factor α (TNFα) in obese adipose tissue suggested that obesity could be an inflammatory status (Hotamisligil et al. 1993). Indeed, adipose tissue is considered as an immune organ. In the SVF of healthy and lean adipose tissue, around 5%–10% cells are immune cells, however, in obese adipose tissue, the ratio of immune cells can be 40%–60% (Hill et al. 2018; Jaitin et al. 2019; Silva et al. 2019; Trim and Lynch 2022). Such chronic inflammation in obese individuals is closely related to the occurrence of insulin resistance and type 2 diabetes. During obesity, hypertrophic adipocytes exhibit increased lipolysis, and upon continuous exposure to hyperinsulinemia, adipocytes can initiate cell cycle and couple with cellular stress to promote their senescence (Ghaben and Scherer 2019). These hypertrophic and senescent cells produce TNFα, interleukin (IL)-1β, IL-6, and monocyte chemotactic protein 1 (MCP-1), and along with inflammatory cells, contribute to the inflammation in adipose tissue (Li et al. 2021; Quail and Dannenberg 2019). Thus, immune cells such as pro-inflammatory macrophages, natural killer cells, neutrophils, CD8+ cytotoxic- and type l T helper (Th1)-lymphocytes, modulate adipocytes and their precursors to influence the adipose tissue remodeling during obesity (Pyrina et al. 2020; Reilly and Saltiel 2017).

Various studies have addressed the impact of cytokines on adipogenesis using the 3T3-L1 cell line and adipose precursors isolated from human or mouse adipose tissue. Many cytokines, such as TNFα, IL-1β, IL-6, IL-17, IL-33, interferon (IFN)-α, IL-4, IL-5, and IL-10 can impair adipogenesis, highlighting that the inflammation triggered by hypertrophic and senescent adipocytes could inhibit the increase of new adipocyte formation, a process to improve the metabolic adaption (Al-Mansoori et al. 2022; Jiang et al. 2019; Kim et al. 2003). However, not all cytokines suppress adipogenesis. IL-7 and IL-34 have been reported to induce adipogenesis (Chang et al. 2014; Lucas et al. 2012; Somm et al. 2005). The most controversial cytokine in regulating adipogenesis is transforming growth factor-β (TGFβ). Some studies demonstrated that TGFβ inhibits PPARγ-C/EBPα complex formation and induces PPARγ phosphorylation, thereby impairing the adipocyte commitment (Ignotz and Massague 1985; Li and Wu 2020). Interestingly, addition of TGFβ at the commitment phase of adipogenesis of human bone marrow- mesenchymal stem cells (BM-MSCs) resulted in an enhancement of adipogenesis, however, the presence of TGFβ in the entire adipogenesis process led to an inhibition of adipogenesis (Elsafadi et al. 2017). These results highlight a double-edged sword effect of TGFβ in adipogenesis. IL-33 is another cytokine that imposes dual roles in regulating adipogenesis. During obesity, the level of circulating IL33 was upregulated (Tang et al. 2021; Zeyda et al. 2013). Administration of recombinant IL-33 could significantly ameliorate diet-induced obesity and related insulin resistance (Kai et al. 2021). This beneficial effect on obesity and related metabolic disorders was found to be related to its suppression on white adipogenesis in vitro (Martinez-Martinez et al. 2015; Miller et al. 2010) and promotion on beiging and thermogenesis in vivo (Li et al. 2020; Odegaard et al. 2016). IL-33-induced beiging relied on group 2 innate lymphoid cells (ILC2s) in WAT which could directly act on adipocytes to enhance uncoupling protein 1 (UCP1) through producing methionine-enkephalin peptides (Brestoff et al. 2015). In obese mice and humans, there was a dramatic reduction of ILC2s in the WAT, a potential link to the limitation of caloric expenditure (Brestoff et al. 2015). Therefore, the failure of enhanced IL-33 to combat obesity and related insulin resistance could be related to the dysfunction of ILC2s. Aging studies on aging revealed the intrinsic defects of ILC2s in aged mice and supplementation of ILC2s from healthy mice, but not IL-33, could prevent old mice from cold-induced lethality (Goldberg et al. 2021). Taken together, detailed understandings of the mechanisms by which cytokines affect adipose tissue at the cellular and molecular levels should help devising new strategies to regulate metabolic homeostasis.

The types and levels of cytokines in adipose tissue are complicated and they dynamically change in the context of stages of obesity. Cytokines in adipose tissue may function at certain stage, or may synergize or antagonize each other when they act on the same adipose precursors. Although the conclusion can be drawn when cytokine such as TNFα was used, the doses applied in in vitro experimental system always relate to a negative effect, like cachexia, a complicated metabolic syndrome related to cancer.

Interestingly, several studies have shown that local pro-inflammatory signals may be necessary for the expansion and remodeling of adipose tissue (Wernstedt Asterholm et al. 2014). Such regulation seems like an adaptive response of the whole body to store excess nutrients. Unlike the response to “chronic” inflammation, transient and acute inflammation in fat depots appears to play a beneficial role in adipogenesis and in maintaining metabolic homeostasis. Upon β3-adrenergic stimulation, the lipolytic products secreted from adipocytes could promote the production of IL-6 and IL-11 from macrophages and endothelial cells. As transient inflammatory signals, these cytokines can promote beige adipogenesis through the Janus kinase/signal transducer and activator of transcription 3 (JAK/STAT3) signaling pathway (Sun et al. 2018). Once chronic inflammation reduced or disappeared, inadequate mobilization in adipose precursors form a vicious circle to promote hypertrophy of adipocytes and disrupt metabolic homeostasis. How a transient local inflammation in vivo is switched on and off to instruct adipose precursors is still an open question.

### The impact of immune regulation on adipogenesis

In lean individuals, the major immune cell populations in adipose tissue are M2 macrophages, eosinophils, ILC2s, Treg cells, B1 cells, γδT cells, and invariant natural killer T cells. Among them, a conduit consisting of eosinophils, and IL-4/13 induced M2 macrophages was found to regulate beige fat mass and thermogenesis (Qiu et al. 2014). The eosinophils and M2 macrophages can be sustained by ILC2s, a kind of innate immune cells for type 2 immune responses through producing IL-5 and IL-13. ILC2s in sWAT activated by IL-33 can promote the proliferation of PDGFRα+ adipose precursors and their commitment to beige adipocytes (Lee et al. 2015). Further studies revealed that ILC2- and eosinophil-derived IL-4 and IL-13 are the key factors in driving the activity of adipose precursors and their beige adipogenesis, thus maintaining metabolic homeostasis (Lee et al. 2015). Similar to that in mice, ILC2s were also identified in human healthy adipose tissue, which is significantly decreased in obese adipose tissue (Brestoff et al. 2015). Targeting IL-33/ILC2s provides a novel approach to treat obesity and related metabolic diseases.

In obese and ageing adipose tissue, an inflammatory pattern is present, with the abundant accumulation of M1 macrophages, neutrophils, mast cells, B2 cells, CD8+ T cells, and Th1 cells. The accumulation of these cells and enhanced levels of related cytokines and chemokines are always linked to obesity and metabolic diseases in human. For example, the expression of C–C motif chemokine ligand 5 (CCL5, as also known as RANTES) and its receptor CCR5 in obese adipose tissue was upregulated and positively correlated with the accumulation of cells expressing CD3 and CD11b. In the T cell and 3T3 L1 co-culture system, activation of T cells suppressed white adipogenesis (Wu et al. 2007). Investigations on ageing mice have found that increased T cells in BAT are senescent and featured with IFNγ. These senescent T cells can suppress the differentiation of adipose precursors into brown adipocytes in both in vivo and in vitro experiment systems (Pan et al. 2021). Taken together, these results demonstrate that the accumulation of pro-inflammatory cells creates a microenvironment with the capacity to impair metabolic homeostasis.

Macrophages are the most abundant population of immune cells in adipose tissue. Their accumulation during obesity development can be maintained by self-renewal as well as continuously derived from peripheral monocytes (Zheng et al. 2016, 2015). Through deciphering immune cell subsets and their dynamic changes in adaption to metabolic stress, it was revealed that macrophages are important in maintaining adipose tissue homeostasis and remodeling (Hill et al. 2018; Jaitin et al. 2019; Silva et al. 2019). Macrophages in adipose tissue can be activated by serum amyloid A3 (Saa3), an adipocyte derived chemokine with the ability to promote adipocyte precursor proliferation and adipose tissue expansion through platelet-derived growth factor alpha polypeptide a (PDGF-aa). More interestingly, the level of Saa3 in mature adipocytes is controlled by the canonical Wnt/β-catenin pathway (Chen et al. 2020). Whole-exome sequencing revealed that gain-of-function mutations in β-catenin increases the risk of obesity (Chen et al. 2020). Therefore, macrophages in adipose tissue release signals to direct the properties of adipose precursors.

### The proinflammatory phenotype of adipose precursors in obese adipose tissue

Adipose precursors in obese individuals possess an inflammatory phenotype, creating curiosities to parse out the heterogeneity of adipose precursors in obese adipose tissue. Indeed, an increase in a subset of adipose precursors featured by high expression of PDGFRα and CD9 is associated with type 2 diabetes. These CD9^high^ adipose precursors gave rise to pro-fibrotic cells and promoted pathological remodeling of WAT to possess obesity properties (Fig. 1). Deletion of CD9 could enhance adipogenesis (Marcelin et al. 2017). Using scRNA-seq to analyze PDGFRβ-expressing adipose precursors, a population of LY6C-CD9-PDGFRβ+ cells were found to spontaneously differentiate into adipocytes, whereas the LY6C+CD9+PDGFRβ+ cells exhibited pro-inflammatory and fibrogenic properties, and thus termed as fibro-inflammatory progenitors (FIPs). These FIPs are located around the blood vessels in vWAT, but not in sWAT, and directly inhibit the differentiation of adipose precursors (Hepler et al. 2018). One possible explanation is that FIPs have an impaired capacity in mitochondrial β oxidation, resulting in a pro-inflammatory phenotype in adipose precursors and lose of lineage commitment to adipocytes (Joffin et al. 2021). Recovering mitochondrial activity in FIPs can rescue their adverse effect on metabolic homeostasis. Meanwhile, another study revealed that the hypoxia inducible factor (HIF1α) signaling was enriched in FIPs suppressed PPARγ and negatively correlated with adipogenesis during diet-induced obesity (Shao et al. 2021a). These studies emphasized the heterogeneity of adipose precursors and their transformation and crosstalk in regulating metabolic fitness and in driving the pathological remodeling of adipose tissue (Ghaben and Scherer 2019; Gonzalez et al. 2018). In addition, Shan et al. found that activation of FIPs during the orchestration of macrophage infiltration in obese adipose tissue is mediated by a down-regulation of ZFP423, a transcriptional suppressor of nuclear factor kappa B (NF-κB) (Shan et al. 2020). The formation of proinflammatory phenotype of FIPs relied on toll like receptor 4 (TLR4), a major receptor for lipopolysaccharide (LPS) and some other stimuli. Various studies have also shown that a group of CD142^high^ stromal cells in adipose tissue impede progenitor cell maturation through a paracrine factor, R-spondin 2 (Rspo2) (Dong et al. 2022; Schwalie et al. 2018). These studies corroborated with the notion of the heterogeneity in the adipose lineage in vivo and their counterbalance in modulating immune status and new adipocyte generation, and provide new insights into the maintenance of metabolic homeostasis.

### The regulation of ECM during adipose tissue remodeling

ECM is a kind of macromolecules produced by many cell types. It is often composed of different biochemical and structural molecules, including collagens, proteoglycans/glycosaminoglycans, elastin, fibronectin, laminins, and several other glycoproteins (Theocharis et al. 2016) and forms an organized three-dimensional ultrastructure to support cell behaviors and functions (Sorokin 2010). It is well-accepted that the stiffness of cell adhesion to distinct ECM components can regulate the commitment of MSCs and their differentiation into adipocytes (Chen et al. 2016; Gumbiner 1996).

During obesity, the remodeling of adipose tissue is accompanied by aberrant ECM deposition, featured by increase in collagen I/IV and laminin α2/4 (Chen et al. 2022). It has been reported that *Lama4* expression is enriched in adipose tissue (Frieser et al. 1997). Mice with *Lama4* deficiency exhibit a significant decrease in fat depots and the resistance to diet-induced obesity and insulin resistance (Vaicik et al. 2014). One explanation for the improved metabolic status in *Lama4*-dedicient mice is an enhancement of beige adipogenesis and energy expenditure (Vaicik et al. 2018). Of note, laminin α4 together with laminin subunits β1 and γ1, form laminin 411, a major component in the basement membrane underling endothelial cells that leads leukocyte extravasation and function switch during inflammation (Sorokin 2010). Therefore, understanding the impact of laminin 411 on immune cells will parse out the role of laminin 411 on the remodeling of obese adipose tissue and related metabolic diseases.

The action of laminins on cells is mediated by integrins. Indeed, various studies have profiled the expression and function of integrins during adipogenesis. Microarray analysis revealed that integrin α5 expression is gradually decreased during adipogenesis, whereas integrin α6 is increased (Liu et al. 2005). Overexpression of integrin α5 increases GTPase activity to preserve the undifferentiated state of adipose precursors, while integrin α6 is critically involved in attachment and migration of adipose precursors and promotes them to reenter the cell cycle and to launch terminal differentiation. An elegant study also found that CD81, a marker of beige adipose precursors, could form a complex with integrins αv/β1 and αv/β5, and mediate the activation of integrin-focal adhesion kinase (FAK) signaling in response to irisin or cold exposure, thus promoting self-proliferation and beige adipogenesis (Oguri et al. 2020). Loss of CD81 exacerbates obesity and insulin resistance. In addition, glycosaminoglycans such as hyaluronic acid (HA) are also important components of ECM. Considering its high biocompatibility, HA is always used as scaffolds in tissue engineering and may facilitate the differentiation of adipose precursors in in vitro and in vivo experiments (Zhu et al. 2019). Interestingly, over production of HA in adipose tissue can improve glucose metabolism through promoting lipolysis in adipocytes, arguing HA synthesis inhibitor should not be a candidate to combat obesity and insulin resistance (Zhu et al. 2021).

During adipose tissue remodeling, ECM components are dynamically regulated. Its degradation mainly relies on matrix metalloproteinases (MMPs) (Bonnans et al. 2014). Previous studies have demonstrated that the mRNA levels of MMP-2, MMP-3, MMP-12, and tissue inhibitor of metalloproteinase (TIMP)-1 are strongly induced in obese tissues, while MMP-7 and TIMP-3 are significantly reduced (Chavey et al. 2003). Similarly, MMPs and TIMPs are involved in human adipose tissue remodeling (Fenech et al. 2015a). Under healthy status, TIMP-3 can promote adipogenesis by cleaving type I collagen and shedding delta like non-canonical notch ligand 1 (DLK1), a transmembrane protein. A soluble form of DLK1 was found to suppress adipogenesis (Fenech et al. 2015b). Furthermore, Chun, et al. showed that membrane-anchored metalloproteinase 1 (MT1-MMP) coordinates adipocyte differentiation in vivo (Chun et al. 2006). In the absence of MT1-MMP, the development of WAT in mice is deficient and causes lipodystrophy (Chun et al. 2006). Meanwhile, in vitro 3D culture experiments demonstrated that MT1-MMP-mediated proteolysis is indispensable for adipocyte maturation through modulating the pericellular collagen stiffness (Gifford and Itoh 2019). Taken together, the quantitative and qualitative changes in ECM regulate the remodeling of adipose tissues under various pathophysiological conditions.

## Adipose tissue remodeling during the pathogenesis of metabolic disorders

### Adipose tissue mass changes during metabolic disorders

Obesity is closely related to metabolic disorders, such as insulin resistance, type 2 diabetes, and ageing. The mobilization of adipose precursors at the early stage of obesity is to adapt to excessive lipid storage. Once the metabolic fitness of adipose precursors loss upon over fat nutrition, the excessive adipocyte hypertrophy would aggregate inflammation in obese adipose tissue and lead to insulin resistance and type 2 diabetes. Recently, obesity has been classified as metabolically healthy obesity (MHO) and metabolically unhealthy obesity (MUO) (Iacobini et al. 2019; Karpe and Pinnick 2015; Wang et al. 2014). The former is mainly characterized by the accumulation of sWAT (buttocks and thighs), with normal insulin sensitivity and adiponectin expression. In contrast, the latter is generally delineated as hypertrophic vWAT increase (abdomen), with significant insulin resistance and high levels of serum triglycerides. To understand the mechanism of fat mass distribution during metabolic diseases, the heterogeneity and plasticity of sWAT and vWAT were compared and analyzed (Fig. 1). In addition to their difference in response to hormones, the adipocyte precursor properties among these two adipose tissues are found to distinct (Schwalie et al. 2018). For example, Myf5, a marker for the precursors of muscle and BAT, can be observed in retroperitoneal sWAT, but not in vWAT (Sanchez-Gurmaches et al. 2012; Shan et al. 2013). The different expression of Myf5 between sWAT and vWAT supports the stronger beige adipogenesis in sWAT upon cold exposure. In contrast, the function of vWAT is to maintain the lubrication and buffering of various organs in the abdominal cavity. Importantly, the internal environment is more complex, which is susceptible to various endogenous and systemic stresses. Apart from the difference of these two adipose tissues in thermogenesis, their expansion also exhibits dissimilar metabolic features. The vWAT maintains efficient lipid metabolism, as their enrichment in glucocorticoids and epinephrine can directly absorb the fatty acids of chylomicrons to produce more free fatty acids. Such process tends to increase lipotoxicity and ectopic fat accumulation. However, sWAT relies on circulating fatty acid redistribution and is insensitive to lipolytic hormones, enabling slow triglyceride turnover to promote fat storage (Karpe and Pinnick 2015). Therefore, fat mass expansion occurred in sWAT is better than that in vWAT, when compared at the same mass amount. Also, in obese mouse models, transplantation of sWAT or removal of vWAT can improve the carbohydrate metabolism caused by obesity (Hocking et al. 2015).

Obesity is not the sole culprit of poor metabolism. When there is a major deficiency in adipose tissue function or development (lipodystrophy), energy storage in adipose tissue does not meet the needs of fatty acid retention, and metabolic disorders occur (Wang et al. 2014). In patients with lipodystrophy, lipids accumulate in secondary peripheral tissues, leading to severe insulin resistance and dyslipidemia (Ghaben and Scherer 2019). This is similar to metabolic disorders in MUO, and even worse, this can be accompanied by severe ectopic fat accumulation in the liver, pancreas, and muscles, as well as systemic metabolic inflammation such as hyperinsulinemia or hypertriglyceridemia (Hussain and Garg 2016). Some congenital or acquired diseases also show differences in fat depots, such as Dunnigan-Kobberling syndrome (Jackson et al. 1998; Peters et al. 1998) and Barraquer-Simons Syndrome (Hussain and Garg 2016). The former is manifested by a loss of fat in limbs but retention in the head, while the latter is the disappearance of sWAT in the upper body but accumulation in the lower body. Therefore, according to the heterogeneity of adipose precursors/adipocytes that distribute in different fat depots, strategies can be developed to recover their normal function. As mentioned above, some disease conditions, like cachexia in patients with advanced cancer, are accompanied by a gradual loss of muscle and fat, which is related to the excessive browning of WAT in rodents and humans. How to prevent the excessive energy consumption and provoke the “sleeping” adipose precursor to replenish adipocytes remains unclear.

### Adipose tissue remodeling during aging

Cellular senescence is a process in which cells are resistant to mitogenic stimulation but metabolically active (Shelton et al. 1999) in response to numerous stressors, including exposure to hypoxia, mitochondrial dysfunction, DNA damage response, and ECM and oncogene activation (Gorgoulis et al. 2019). In 2015, several studies demonstrated that adipocyte senescence was initiated by an elevation in DNA damage (Chen et al. 2015). During ageing, the properties of adipose precursors are altered. It has been demonstrated that the self-renewal and differentiation of adipose precursors decline along with ageing (Kirkland et al. 1990). Also, compared to mature adipocytes, aged adipocytes exhibit an increase in senescence and a significant enhancement in the expression of pro-inflammatory cytokines and ECM components (Zoico et al. 2020).

Aging is closely associated with the redistribution of adipose tissues, with reduced sWAT and enhanced vWAT (Kuk et al. 2009). In aged adipose tissues, the infiltration of inflammatory cells and the enlargement of LDs in adipocytes are severer (Tabula Muris 2020). An inadequate capacity to store lipids in hypertrophic adipocytes and a subsequent conspicuous lipid deposition in ectopic fat depots result in systemic lipotoxicity, a process that contributes to adverse metabolic outcomes (De Carvalho et al. 2019). Healthy adipose tissues release a variety of molecules that maintain systemic energy balance, but such secretion is impacted by aging. In aged vWAT, the expression of catecholamine-degrading enzymes in adipose tissue macrophages is elevated, thereby blocking noradrenaline-induced lipolysis in vWAT (Camell et al. 2017). However, in humans, such age-related function of monoamine catabolism in WAT is attributed to the upregulation of catecholamine degradation in adipocytes rather than in macrophages (Gao et al. 2020). In addition, aged WAT showed a downregulation of C/EBPα expression and an upregulation of the markers related to endoplasmic reticulum stress (Ghosh et al. 2016; Karagiannides et al. 2001).

Distinct from WAT, BAT mass is relatively stable in postnatal development. During ageing, BAT begins to decline the abundance of mitochondria, increase the lipid storage, and decrease in the utilization of intracellular energy blocks (Sellayah and Sikder 2014), revealing an impaired ability in thermogenesis (Saely et al. 2012). This impairment in thermogenesis was related to an increase in mitochondrial DNA mutations and a reduction of biogenesis and oxidative phosphorylation (Zoico et al. 2019). Posteriorly, the sympathetic nervous system also plays a key role in regulating BAT. Several studies have clearly shown that in older lean subjects, both sympathetic drive and BAT activity were lower as compared to younger lean and obese men (Bahler et al. 2016). However, compared to the investigations on BAT, there are relatively few studies on beige adipose tissue. Qiang et al. suggested that sirtuin-1 (SIRT1) as an important target in adipose tissue could promote the browning of WAT through enhancing the interaction between PPARγ and PRDM16 (Becerril et al. 2013). A reduction in SIRT1 level can be observed in aged human, and microRNA-34a has been found to be a direct regulator of SIRT1 (Choi and Kemper 2013). Interestingly, miRNA-34a suppresses the thermogenesis in obesity through partially regulating SIRT1 and fibroblast growth factor 21 (FGF21), another muscle factor recently discovered in browning of sWAT (Fisher et al. 2010). Therefore, deciphering the mechanism of aged adipose tissue in energy control and metabolism homeostasis could provide new insights into designing better strategies to combat the dysfunction of adipose tissue and related metabolic conditions.

## Conclusions and perspectives

The advent of scRNA-seq technology and lineage tracing models have revolutionized the understanding of adipose precursors and their capabilities in remodeling adipose tissue during various pathophysiological conditions. Their heterogeneity is not limited to generate new adipocytes with distinct distributions and properties in conserving energy, but also is related to their capabilities in orchestrating signals to regulate tissue microenvironments, such as inflammation and fibrosis. Also, it is essential to apply the knowledge derived from human adipose tissue, adipose precursors and in vitro experiment models to deconstruct the cellular and molecular basis of adipose precursors and their interaction with tissue microenvironment components under various pathophysiological conditions. This information could lead to the establishment of powerful strategies to combat ageing and chronic metabolic diseases. In terms of obesity related insulin resistance and diabetes, rationally mobilizing adipose precursors to increase the number of adipocytes, either white or beige adipocytes could be valuable in improving the metabolic fitness. In addition, turning excess white adipose tissue into energy-burning brown/beige adipose tissue could be suitable for MUO, while increasing the adipose tissue mass and converting excessive energy-intensive adipocytes into white adipocytes are also expected to benefit patients suffering from cachexia and lipoatrophy. Although we cannot customize healthy adipose precursors according to individual demands, thoroughly appreciating the heterogeneity of adipose precursors and their interaction with various components in the tissue microenvironment should support the development of novel strategies to remodel adipose tissue to maintain metabolic homeostasis.

## Data Availability

Not applicable.

## Abbreviations

WAT: White adipose tissue
BAT: Brown adipose tissue
LD: Lipid droplet
scRNA-seq: Single cell RNA-sequencing
MSCs: Mesenchymal stem cells
Wt1: Wilm tumor gene 1
vWAT: Visceral WAT
sWAT: Subcutaneous WAT
SVF: Stromal vascular fraction
Myf5: Myogenic factor 5
Pref1: Preadipocyte factor 1
Sox10: SRY-box transcription factor 10
Wnt1: Wnt family member 1
Sca-1: Stem cell antigen-1
αSMA: α-smooth muscle actin
PDGFRα/β: Platelet-derived growth factor receptor α/β
PRDM16: PR domain-containing protein 16
DPP4: Dipeptidyl peptidase 4
ICAM1: Intercellular cell adhesion molecule 1
Aregs: Adipogenesis-regulatory cells
ABCG1: ATP binding cassette subfamily G member 1
TMEM26: Transmembrane protein 26
BMP2/4/7: Bone morphogenesis protein 2/4/7
SMAD4: SMAD family member 4
EBF1/2: Early b-cell factor 1/2
ZFP423/521: Zinc finger protein 423/521
CREB: cAMP-response element-binding protein
C/EBPα/β/δ: CCAAT/enhancer-binding protein α/β/δ
PPARα/γ: Peroxisome proliferator-activated receptor α/γ
EWSR1: Ewing sarcoma breakpoint region 1
YBX1: Y-box-binding protein 1
PGC-1α: Peroxisome proliferator-activated receptor γ coactivator 1α
IRF4: Interferon regulatory factor 4
SCD1: Stearoyl-CoA desaturase 1
ECM: Extracellular matrix
TNFα: Tumor necrosis factor α
IL: Interleukin
MCP-1: Monocyte chemotactic protein 1
Th1: Type l T helper
IFNα/γ: Interferon α/γ
TGFβ: Transforming growth factor β
BM-MSCs: Bone marrow- mesenchymal stem cells
ILC2s: Group 2 innate lymphoid cells
UCP1: Uncoupling protein 1
JAK/STAT3: Janus kinase/signal transducer and activator of transcription 3
CCL5: C–C motif chemokine ligand 5
CCR5: C–C motif chemokine ligand 5 receptor
Saa3: Serum amyloid A3
PDGF-aa: Platelet-derived growth factor alpha polypeptide a
FIPs: Fibro-inflammatory progenitors
HIF1α: Hypoxia inducible factor 1α
NF-κB: Nuclear factor kappa B
TLR4: Toll like receptor 4
Rspo2: R-spondin 2
FAK: Focal adhesion kinase
LPS: Lipopolysaccharide
HA: Hyaluronic acid
MMPs: Matrix metalloproteinases
TIMP: Tissue inhibitor of metalloproteinase
DLK1: Delta like non-canonical notch ligand 1
MT1-MMP: Membrane-anchored metalloproteinase 1
MHO: Metabolically healthy obesity
MUO: Metabolically unhealthy obesity
SIRT1: Sirtuin-1
FGF21: Fibroblast growth factor 21
