# Correction: Characteristic and fate determination of adipose precursors during adipose tissue remodeling

**DOI:** 10.1186/s13619-023-00166-7

**Published:** 2023-05-16

**Authors:** Jiayin Ye, Cheng Gao, Yong Liang, Zongliu Hou, Yufang Shi, Ying Wang

**Affiliations:** 1 CAS Key Laboratory of Tissue Microenvironment and Tumor, Shanghai Institute of Nutrition and Health, University of Chinese Academy of Sciences, Chinese Academy of Sciences, 320 Yueyang Road, Shanghai, 200031 China; 2 Key Laboratory of Tumor Immunological Prevention and Treatment of Yunnan Province, Kunming, 650000 Yunnan China; 3 The Third Afliated Hospital of Soochow University and State Key Laboratory of Radiation Medicine and Protection, Institutes for Translational Medicine, Soochow University, 199 Renai Road, Suzhou, 215123 Jiangsu China

**Correction: Cell Regen 12, 13 (2023)**

**https://doi.org/10.1186/s13619-023-00157-8**

Following publication of the original article (Ye et al. 2023), the authors reported that the “Competing interests” section needed to be updated.

The original version was:

The authors declare no competing interests.

The updated version is:

Yufang Shi is a member of the Editorial Board for Cell Regeneration. He was not involved in the journal’s review of, or decisions related to, this manuscript.

The original article (Ye et al. 2023) has been corrected.
